# Operational accuracy and comparative persistent antigenicity of HRP2 rapid diagnostic tests for *Plasmodium falciparum *malaria in a hyperendemic region of Uganda

**DOI:** 10.1186/1475-2875-7-221

**Published:** 2008-10-29

**Authors:** Daniel J Kyabayinze, James K Tibenderana, George W Odong, John B Rwakimari, Helen Counihan

**Affiliations:** 1 Malaria Consortium Africa, Plot 2, Sturrock Road, P O Box 8045, Kampala, Uganda; 2 London School of Hygiene and Tropical Medicine, Keppel Street, London, WC1E 7HT, UK; 3 Soroti Regional Referral Hospital, P.O. Box 289 Soroti, Uganda; 4 Malaria Control Programme, Ministry of Health, P.O. Box 7272, Kampala, Uganda; 5 Malaria Consortium, Development House, 56-64 Leonard Street, London, EC2A 4LT, UK

## Abstract

**Background:**

Parasite-based diagnosis of malaria by microscopy requires laboratory skills that are generally unavailable at peripheral health facilities. Rapid diagnostic tests (RDTs) require less expertise, but accuracy under operational conditions has not been fully evaluated in Uganda. There are also concerns about RDTs that use the antigen histidine-rich protein 2 (HRP2) to detect *Plasmodium falciparum*, because this antigen can persist after effective treatment, giving false positive test results in the absence of infection. An assessment of the accuracy of Malaria Pf™ immuno-chromatographic test (ICT) and description of persistent antigenicity of HRP2 RDTs was undertaken in a hyperendemic area of Uganda.

**Methods:**

Using a cross-sectional design, a total of 357 febrile patients of all ages were tested using ICT, and compared to microscopy as the gold standard reference. Two independent RDT readings were used to assess accuracy and inter-observer reliability. With a longitudinal design to describe persistent antigenicity of ICT and Paracheck, 224 children aged 6–59 months were followed up at 7-day intervals until the HRP2 antigens where undetectable by the RDTs.

**Results:**

Of the 357 patients tested during the cross-sectional component, 40% (139) had positive blood smears for asexual forms of *P. falciparum*. ICT had an overall sensitivity of 98%, a specificity of 72%, a negative predictive value (NPV) of 98% and a positive predictive value (PPV) of 69%. ICT showed a high inter-observer reliability under operational conditions, with 95% of readings having assigned the same results (*kappa *statistics 0.921, p < 0.001).

In children followed up after successful antimalaria treatment, the mean duration of persistent antigenicity was 32 days, and this duration varied significantly depending on pre-treatment parasitaemia. In patients with parasite density >50,000/μl, the mean duration of persistent antigenicity was 37 days compared to 26 days for parasitaemia less than 1,000/μl (log rank 21.9, p < 0.001).

**Conclusion:**

ICT is an accurate and appropriate test for operational use as a diagnostic tool where microscopy is unavailable. However, persistent antigenicity reduces the accuracy of this and other HRP2-based RDTs. The low specificity continues to be of concern, especially in children below five years of age. These pose limitations that need consideration, such as their use for diagnosis of patients returning with symptoms within two to four weeks of treatment. Good clinical skills are essential to interpret test results.

## Background

Prompt and accurate diagnosis of malaria is the key to effective case management and a major component of Uganda's malaria control strategy. Clinical diagnosis is the least expensive and most widely practised method and the one generally used for self treatment. However, the overlapping of malaria symptoms with other tropical diseases impairs its specificity and can promote the indiscriminate use of anti-malarials and compromise quality of care for patients with non-malarial fevers in endemic areas [1]. Therefore the accuracy of malaria diagnosis can be greatly enhanced by combining clinical and parasite-based findings.

Conventional light microscopy is the "gold standard" for routine parasite-based diagnosis because it is sensitive, inexpensive to perform, can differentiate malaria species and quantify parasite load. Microscopy requires well-trained technicians, a microscope in good working order and a well-functioning quality assurance system. The challenges for governments to ensure that such prerequisites are in place mean that microscopy is unavailable at many peripheral health facilities in resource limited settings, where most cases are managed in the public health sector. Rapid diagnostic tests (RDTs) require less expertise to be conducted correctly, and non-specialized staff can be quickly trained to use them. Malaria diagnostic accuracy may be strengthened by the use of RDTs where microscopy is not available or feasible to maintain.

Uganda changed its malaria drug policy in 2004 from chloroquine plus sulphadoxine-pyremethamine (CQ+SP) to artemisinin-based combination therapy (ACT) using Coartem™ (artemether-lumefantrine) as the first-line treatment. ACT is a more expensive treatment than the previous drug combination of CQ+SP, and the Ministry of Health (MoH) is thus keen to shift malaria diagnosis to become parasite-based rather than clinical. This is to ensure that only confirmed cases of malaria receive anti-malarials and differential diagnosis is utilized to identify the cause of fever in cases giving a negative result for malaria. In this way, the quality of care of fever cases will be greatly improved and the wastage of anti-malarials will be minimized.

A review of studies evaluating diagnostic tools for malaria found very high accuracy and reliability of RDTs [2]. The tests are cost effective and can lead to cost saving when used instead of clinical diagnosis and treatment [3]. Malaria antigens currently targeted by RDTs are histidine rich protein 2 (HRP2), which is unique to *Plasmodium falciparum*, Plasmodium lactate dehydrogenase (pLDH), and Plasmodium aldolase. The current policy guideline in Uganda recommends use of HRP2, due to the very high proportion of *P. falciparum *infections [4].

HRP2-based RDTs are very sensitive in detecting *P. falciparum *infections, are heat stable[5] and generally cost less than many of the other types of RDTs [6]. However, persistent antigenicity in blood after treatment can reduce the specificity of these RDTs in previously infected individuals, especially in highly endemic areas. In these areas, the duration of persistent HRP2 varies widely and can be four weeks or longer [7-9]. Low specificity and false positive RDTs results leads to misuse of anti-malarials, and consequently increased drug presure and indirectly contributes to drug resistance in such cirmustances.

Currently, there is limited information on the accuracy of Malaria Pf™ ICT (ICT) marketed by ICT Diagnostics, South Africa. The data available [3,7,10-16] is based on three other RDTs that have all evolved from the one original product. Although ICT is very similar to these three RDTs, it has a different format and nitrocellulose strip and is manufactured at a different site – factors recognized to have a potential influence on the test's sensitivity and specificity. In addition, ICT detects *P. falciparum *only, unlike previously evaluated RDTs that detect all four human malaria species. There have been a number of other studies on malaria RDTs done in Uganda [17-21].

This study assesses the operational accuracy and ease of use (inter-observer and intra-test reliability) of ICT in a district hospital setting. It also describes persistent HRP2 antigenicity of both ICT and Paracheck Pf (another HRP2-based RDT) in children aged 6–59 months treated for malaria in a hyperendemic setting in Uganda.

## Methods

### Study site

The study was conducted as two independent serial components. A cross-sectional assessment was performed to determine the accuracy of ICT for *P. falciparum *infection using microscopy as the gold standard. In addition, a longitudinal design was used to describe persistent antigenicity using two HRP2 tests (ICT and Paracheck). Both components were undertaken in Soroti Regional Referral Hospital, Uganda. Soroti town is 347 kilometres north of the capital Kampala and is in an area of hyperendemic malaria with a parasite prevalence of 85% in children below nine years of age [4]. The out-patient department (OPD) handles, on average, 18,000 suspected malaria cases per year, of which 50% have blood smears performed; the majority of these (60%) are positive for malaria (MoH Uganda, unpublished data). The study protocol was approved by the Uganda National Council of Science and Technology.

### Study participants

For both study components, consecutive patients presenting to the OPD were screened for symptoms/history suggestive of malaria and evaluated for eligibility into the study. During the cross-sectional component, patients who fulfilled the following criteria were enrolled: 1) aged six months and above; 2) history of fever in the last 24 hours or axillary temperature ≥ 37.5°C; 3) no evidence of a concomitant febrile illness; 4) provision of informed consent; 5) no danger signs or evidence of severe malaria.

For the longitudinal component, only children aged 6–59 months and residing within seven kilometres from the hospital were recruited. The additional inclusion criteria were as follows: 1) *Plasmodium falciparum *monoinfection on blood smear; 2) positive RDT test results; 3) no evidence of a concomitant febrile illness; 4) no danger signs or evidence of severe malaria; 5) provision of informed consent including extended follow-up; and 6) negative blood smear for asexual-stage peripheral parasitaemia on day-3 after anti-malarial therapy.

### Study procedures

#### Cross-sectional component procedures

At screening, patients or guardians were asked about prior anti-malarial use and presence of common symptoms. Weight and axillary temperature were measured and a physical examination performed and patients that provided consent were referred to the laboratory for investigation.

In the laboratory, blood was obtained by finger prick for thick and thin blood smears, for RDT and a drop was taken on a filter paper for possible future molecular analysis.

Blood smears were stained with 10% Giemsa for 30 minutes. Thick smears were examined for parasites including gametocytes and parasite densities were determined by counting the number of asexual parasites per 200 white blood cells (WBC), or per 500 WBC if the parasite count was less than 10 parasites per 200 WBC, assuming a WBC count of 8,000/μl. A slide was considered negative if no parasites were seen after reviewing 100 high power fields. Thin smears were read to determine the *Plasmodium *species. Patients were managed based on microscopy results and national treatment guidelines. Medication was dispensed at a general OPD pharmacy unsupervised by the study team.

A RDT result was interpreted as positive when both the test line and control line showed pink, negative when only the control line showed pink or invalid when the control line did not appear regardless of the test line. Two independent readings were graded based on visual assessment as "strong" or "faint positive" for reactive tests and "negative" for non-reactive ones. A test result was graded "strong" if the test line was as intense as the control line and a "faint" result was a line that could only be seen in good light. The reason for this distinction on the positive line intensity is that a strong result is very clear, but faint positive lines can easily be missed in situations where the lighting is poor or the operator has poor eyesight. Grading was also used to determine the source of variability in results reported by two independent readers. Any invalid RDT tests were not repeated.

#### Longitudinal component procedures

During the longitudinal component of the study, patients were evaluated simultaneously using Paracheck and ICT RDTs at screening and on every follow-up day. Only patients with a negative day-3 smear were enrolled for the longitudinal component. Enrolled patients were asked to return for follow-up on day-7 and every subsequent seven days or any other day that they felt ill. Follow-up evaluation consisted of taking finger prick blood for RDT tests and blood smears. Enrolled patients with complaints were also clinically evaluated and managed. The follow-up period ended when an outcome was determined as either smear-positive or RDT-negative. Patients with positive smears were referred to the clinician for evaluation and treatment.

#### Operational quality control

Local hospital staff were involved in the day-to-day conduct of the study. Both laboratory and clinical teams were trained to perform the RDT. The training was followed with a pilot study of about 20 patients to ensure that all study procedures were clearly followed and that the study did not cause any unnecessary interference to the normal functioning of the clinics and the laboratory. Laboratory technologists were assigned work according to hospital duty register. All study slides were read by first and second microscopists who were blinded to each others' results. The same second microscopist was maintained throughout the longitudinal component to ensure consistency. Reading were considered discordant for the following scenarios: 1) positive/negative discordance for asexual stages; 2) species discordance; 3) asexual discordance of more than 50% difference between the parasitaemia; 4) positive/negative discordance for gametocytes. A third, external microscopist, unaware of the first two results, resolved any discordant results and read a further random 10% for external quality control purposes. Similar to microscopy, RDT cassettes were labelled and read by two laboratory technologists independently 15 minutes after adding buffer. Temperature and humidity for the storage conditions for RDTs during study period were monitored using Tinytag™ Data Loggers  with an alarm set at 37°C.

### Study outcome measures

The outcome measures were RDT and microscopy results for the cross-sectional component. During the longitudinal component, the possible outcomes at each follow-up evaluation were; 1) positive RDT and negative smear interpreted as persistent antigenicity; 2) both RDTs and smear negative interpreted as cleared antigen; and 3) positive RDT and positive smear interpreted as a recurrence of asexual parasites (possible recrudescence or re-infection). Patients with persistent antigenicity at any time point were given a 7-day follow-up appointment. Patients were excluded from further follow-up if they withdrew consent or both RDT readings were invalid or not interpretable.

### Sample size estimation

To determine accuracy of ICT, sample size was calculated considering a sensitivity of 90% and a prevalence of malaria in the study population of 50% (among children under 10 years). Using the nomogram by Carley *et al *[22], at a precision of 5% and an alpha error of 0.05, a sample population of 300 subjects plus an additional 10% to allow for invalid and unclear results was estimated.

For the longitudinal component, to describe and determine predictors of persistent antigenicity, a sample of 224 participants that completed follow-up showed >80% power of detecting a difference between low parasite density (less than 1,000/μl), and high (>50,000/μl) day-zero parasitaemia.

### Statistical methods

Data were entered and verified using EpiData 3.01 and analysed using SPSS version 12.0 . Overall agreement, as a measure of reliability of RDT readings was calculated using a kappa statistics. Using microscopy as the reference, sensitivity of the RDT was measured as the proportion of RDT positives over the total positives determined by microscopy and specificity as proportion of RDT negatives over the total negatives determined by microscopy. The positive predictive value (PPV) is the number of RDT true positives divided by the number all RDT positive test results (true positive plus false positives). The negative predictive value (NPV) is the number of RDT true negatives divided by the number all RDT negative test results (true negatives plus false negatives).

Duration of persistent antigenicity was measured as time in days, when RDT remains positive after treatment and the blood slide remains negative. Data from patients with recurrent parasitaemia were included in the analysis of persistent antigenicity until the day the microscopy smear became positive and an outcome measure of the previous time-points assigned. Inter observer reliability of ICT and intra-test reliability of ICT and Paracheck were calculated using *Kappa *statistics. For analysis purposes, day-zero parasite density was categorized into three groups; <1,000/μl, 1,000–50,000, and > 50,000/μl. The mean duration was determined for persistent antigenicity (survival analysis techniques) and compared equality of survival distribution for categorized day-zero parasite density using log rank test statistics.

## Results

### Study participants

The study was conducted between October 2006 and September 2007 in Soroti Hospital Out-Patients Department (OPD) in two components. During the cross sectional component, 364 patients were recruited into the study, but seven patients were excluded from this analysis because of unreadable blood smears. Of the 357 patients evaluated, the median (IQR) age was 11 (1–28) years, 46% were under the age of five, and 60% female. A total of 139 (39%) had positive blood smears for asexual forms of *P. falciparum*, predominately in the children under five years, where 102 (73%) were positive (Table 1).

**Table 1:** Baseline characterizes of participants in the HRP2 RDT evaluation study, Soroti Hospital, Uganda, October 2006 and September 2007

	**Cross-sectional component**		**Longitudinal component**	
**Characteristics**	**Microscopy result (n = 357)**		**Follow-up events (= 310)**	

	Positive (n = 139)	Negatives (n = 218)	Completed (n = 224)	Lost (n = 86)

Female, n(%)	64(40%)	145(65%)	122(55%)	46(54)

Age in years, median (IQR)	1.0(0.8–6.0)	23(3.0–34.0)	1.3(0.8–2.0)	1.2(0.7–1.6)

Children less than 5 years, n(%)	102 (73)	61(28)	All	All

Temperature °C, mean (SD)	38.0(1.3)	36.8(0.9)	38.0 (1.2)	37.8 (1.2)

Anti-malarial use in previous 2 weeks	54(39%)	103(65%)	156(70)	58(67)

Parasite density/μl, geometric mean	60,074	-	31,460	29,195

Gametocytes on day-zero, n (%)	7(5%)	-	9(4%)	4(5%)

Weight in kilograms, mean(SD)	-	-	10.0(2.7)	9.4(2.4)

IQC, interquartile range: SD, standard deviation

**Figure 1:**
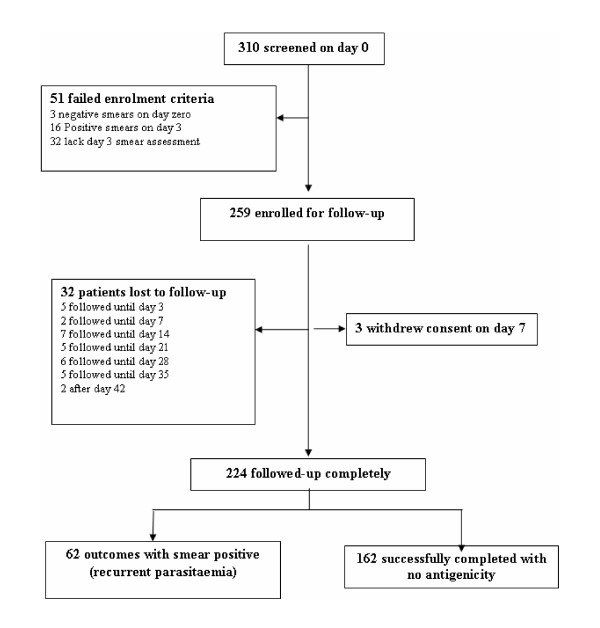
**Longitudinal component patient profile**. Longitudinal component patient profile showing screened patients, smear and rapid diagnostic test (RDT) results and the number that were followed up. At the beginning of the longitudinal component to evaluate persistent antigenicity of HRP2 RDTs, 259 children were enrolled on day-3. 224 remained in the study until an outcome was determined (at the end of the follow up when either the RDTs were negative or smear was positive due to recurrent parasitaemia).

During the longitudinal component to describe persistent antigenicity, a total of 310 febrile children with *P. falciparum *monoinfection were screened with both ICT and Paracheck RDTs on day-zero. On day-3, participants were evaluated to determine anti-malarial treatment response. Of the 310 patients screened on day-zero, 51(16.4%) were not enrolled in the follow-up component due to the following reasons: sixteen still had asexual parasitaemia (early treatment failures); three children with negative smears on day-zero had been included in violation of the protocol, while 32 children who missed day-3 evaluation were excluded because anti-malarial treatment response could not be determined in time. Out of the 259 children successfully enrolled for follow-up after day-3 evaluation, three patients withdrew consent citing unwillingness to continue with a hospital based follow-up and thirty-two others failed to honour appointments during the course of follow-up (Figure 1).

### Operational accuracy of ICT

The ICT had a sensitivity of 98% (95% CI: 94–99) and specificity of 72% (95% CI: 65–77). The specificity of ICT was significantly lower in the <5 years at 54% (95% CI: 41–67) compared to 78% (95% CI: 71–85) in the older age group. Overall, ICT test had a NPV of 98% (95% CI: 95–100) and PPV of 69% (95% CI: 62–75). The 78% (95% CI 58–70) PPV in the under fives is significantly higher than 51% (95% CI 39–64) in the above fives (Table 2). Of the 198 positives ICT test, 62(31%) were false positive. There were 159 negative ICT results of which three were false negatives. Two out of the three false negative results had a low parasite density of 32 and 440/μl, while the third false negative RDT had a high parasite density of 70,400/μl.

**Table 2:** Accuracy of malaria P.f. ™ ICT rapid diagnostic test for the detection of Plasmodium falciparum infection in 357 patients attending out-patient clinic in Soroti Hospital, Uganda

**Accuracy measure**	**Overall (n = 357)**	**< 5 years (n = 163)**	**≥ 5 years (n = 194)**
	**% (95% CI)**	**% (95% CI)**	**% (95% CI)**
Sensitivity	98 (94–99)	98 (93–100)	97(86–100)
Specificity	72 (65–77)	54 (41–67)	78 (71–85)
Positive predictive value	69 (62–75)	78 (58–70)	51 (39–64)
Negative predictive value	98 (95–100)	94 (81–99)	99 (96–100)
ICT true positives, n(% pos)	136(69%)	36(51%)	100(78)
ICT true negatives, n(% neg)	156(98%)	123(99%)	33(94%)

### Reliability of HRP2 tests

For the cross-sectional component of the study, the results of the first and second ICT readings were highly comparable with 336/354 (95%) concordant reading (*kappa *statistics 0.921, p < 0.001), showing a very high operational test reliability.

During the longitudinal component of the study, all tests were performed using ICT and Paracheck RDTs. Results where graded as "strong" positive, "faint" positive and negative. Comparing ICT and Paracheck paired tests done for the same patient during the follow-up component showed 1488/1559(95%) concordance between the two RDT results. The major difference between the two HRP2 tests was in terms of line intensity as "strong" versus 'faint" positives. There were 17 discordant pairs, for which ICT showed 14 as negative while Paracheck showed "faint positive". Two "faint positive" ICT tests were negative on Paracheck. Only one negative ICT showed "strong positive" with Paracheck. Overall, there were eight invalid ICT tests (Table 3). When all strong and faint positives where grouped as positives, diagnosis interpretations for both test results showed 1542/1559 (99%) concordance. The results showed very high intra-test reliability (*kappa *statistics = 0.98, p < 0.001) and further analysis below is presented simply as HRP2 results and does not distinguish between the two RDTs.

**Table 3:** Comparison of two HRP2 rapid diagnostic tests in a longitudinal follow-up of 310 children with *P. falciparum *monoinfection in Soroti Hospital, Uganda

**RDT type**	**Paracheck**				
ICT		Strong Positive	Faint Positive	Negative	Invalid
	
	Strong Positive	904	20	0	0
	Faint Positive	23	424	2	2
	Negative	1	14	159	0
	Invalid	3	4	0	1

### Persistent HRP2 antigenicity

For all the 310 children screened on day-zero, there was no significant difference at baseline of gender, age, temperature, parasite density, presence of gametocytes or recent anti-malarial use between those who completed follow-up and the ones that did not (Table 3). At the end of the follow-up period, 224/259 (86.4%) children had been assigned an outcome for persistent antigenicity (Figure 1).

**Figure 2:**
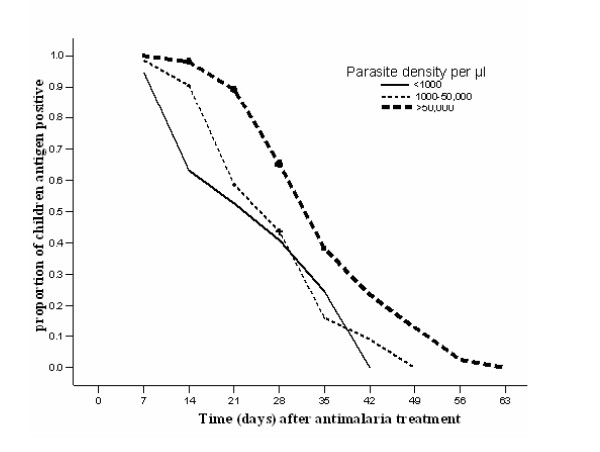
**Proportion of children with persistent antigen in Soroti, Uganda**. Proportion of children with persistent antigen positive HRP2-RDT at clinic visits, stratified by day-zero parasite density at < 1,000/μl, 1,000–50,000/μl and > 50,000/μl. 224 children were followed up with censoring for those with recurrent parasitaemias. The log rank test statistics for equality of survival distributions was 21.93 (p < 0.001).

Of these, 162 (72%) were followed up until the antigenicity was no longer detectable while 62 (28%) had recurrent peripheral parasitaemia detected by microscopy during the course of the follow-up time at which point they were no longer followed-up. Persistent antigenicity was documented in 98% of the cases on day-7 and this decreased to 14% by day-35 (Table 4). The overall mean duration of persistent antigenicity was 32 days (95% CI: 31–34) and median of 35 (95% CI: 33–37) days. Pre-treatment parasite density predicted duration of antigenicity after adjusting for age, temperature and prior anti-malarial use. In patients with pre-treatment parasite density >50,000/μl, the mean duration of persistent antigenicity was 37 days compared to 26 days for parasitaemia less than 1,000/μl (log rank 21.9, p < 0.001)(Figure 2). The results remained statistically significant when parasite densities were reclassified as well when patients lost to follow-up were included.

**Table 4:** Proportion of children with persistent HRP2 antigenicity during follow-up in Soroti, Uganda

**Duration(days) of follow-up**	**Number that turned negative for antigen test**	**% Persistent antigen positive (n = 162)**	% Persistent antigen positive (n = 224)*
3	0	100	100
7	5	97	98
14	14	88	84
21	41	63	60
28	35	41	35
35	38	18	14
42	14	9	7
49	10	3	2
56	4	1	0
63	1	0	0

* Inclusive of children with recurrent parasitaemia.

## Discussion

The study evaluated the performance of the ICT rapid diagnostic test relative to microscopy in an operational setting. The results of the study demonstrate that ICT test is an accurate and reliable method of diagnosing *P. falciparum *malaria in the context of microscopy. The very high sensitivity of 98% implies that the majority of malaria case among this population would be accurately diagnosed, giving the clinician the confidence that true cases of malaria are rarely missed. Most false negatives are linked to low parasitaemias which are unlikely to be the primary cause of illness. There were three false negatives results, two with parasite density <1,000/μl and one with parasite density > 5,000/μl. The last result where the parasite density was high, has been described by other investigators as a very rare event which may be caused by a prozone effect or by the presence of a mutation or deletion within the HRP2 gene [23]. Two studies have reported cases of false negatives at very high parasitaemia with both a pLDH-based test and a HRP2 test with repeated testing [24,25], but again in very low numbers – 1 case out of 58 and 2 cases out of 196 patients respectively.

Related to the high sensitivity, IC T demonstrated a very high NPV in this study population that allows a clinician to confidently diagnose negative-test patients as non malaria patients and look for other causes of fevers. The averted non-malarial treatments would lead to cost saving in the health sector and improved quality of care [3,26]. The results showed a relatively low specificity especially in the under fives where it was 54%. Having a lower specificity, which leads to over diagnosis and treatment, was considered to result into less serious consequences than a low sensitivity in the context of clinical diagnosis. It is worth noting however, that the PPV in the under 5s was 78% which might be perceived as surprisingly high with such a low specificity and is linked to the high prevalence of malaria in this population. It is very likely that the low specificity has resulted from persistent antigenicity in patients who had recently been successfully treated for malaria.

ICT was found to be a reliable test with a high inter-reader and intra-test reliability Only 8 (2%) discordant records were assigned different interpretations as "faint positives" versus negative results. There was a high level of result concordance between ICT and Paracheck tests during longitudinal component. The two HRP2 tests were of comparable accuracy and performance in an operational setting.

This implies that given minimum training, it is feasible for field workers to interpret and report consistent results.

The study also described the duration of ICT false positivity due to persistent antigenicity and its relationship to pre-treatment *P. falciparum *parasite density in Ugandan children. Overall, persistence of HRP2 antigenicity was detectable in the majority of children at three weeks following successful treatment. The mean duration of 32 days is particularly worrying because HRP2 persistent antigenicity reduces the accuracy of the RDT test. This is more of concern in hyperendemic areas, where frequent malaria infections mean that children are likely to have the antigen in their blood even if their fever is not caused by malaria. There is a danger of over-diagnosis of malaria based on the interpretation of HRP2 test results. Misdiagnosis results in poor quality of care as the appropriate illness may not be addressed in time. It may also result in misuse of anti-malarials, poor resource allocation and, clinicians doubting the efficacy of the medicine used. Clinical skills in taking history and eliciting signs are essential when interpreting RDT results in an effort limit misuse of anti-malarials. Misuse of anti-malarial contributes to drug pressure and spreading of drug resistant *P. falciparum *[27], resulting in increased malaria-specific mortality, especially in children[28].

Previous studies from Asian countries have also reported persistent antigenicity after malaria clinical cure and the levels reported have varied widely, with ranges of 29–65% on day-7 [7,8,29-31]. In this study, longer durations of persistent antigenicity were reported and were shown to be associated with high parasite density. In general parasite densities are significantly higher in Africa than in Asia and may explain why similar studies in Asia have not found such a high level of prolonged false positivity. These results show that the high parasitaemia is directly proportional to persistent antigenicity and support what Swarthout and others found in the DR Congo, another hyperendemic region in Africa[8].

The causes of persistent antigenicity after malaria therapy are still debatable. Previous studies have listed causes of persistent antigenicity as parasite density levels below detection threshold for microscopy[32], delayed clearance of circulating antigens and, presence of gametocytes that also produce the antigen [9]. The most plausible explanation in this study is the high pre-treatment parasitaemia. Patients with high parasite densities have large amounts of HRP2 antigen secreted in proportion to the parasite numbers [33] and to their stage of development [34]. Elevated concentrations of HRP2 evidently take longer to eliminate [29] which seems to be supported by the findings of this study. The observed 28% recurrence of parasitaemia during follow-up may be a reflection of resistant parasites that persisted as sub-patent parasitaemia (<50 parasites/μl) or new infections. This raises the question of whether the positive results with the RDTs were actually false positives or the RDT was correctly detecting these sub-patent infections[32]. However, a recent study in Uganda found 29% risk of recurrent parasitaemia of which 16% was due to recrudescence after treatment with artemether-lumefantrine (AL) over a 42 day follow-up period in a similar setting [35]. Since this current study also reported a similar proportion of recurrence of parasitaemia in a hyper-endemic area, the high proportions reported here as result of persistent antigenicity are possible and not a mere reflection of recrudescence or re-infection.

The study participants were drawn from a pool of all persons suspected of having malaria, regardless of history, clinical status, demographic characteristics or other factors that may affect sensitivity and specificity in a routine health clinic. Inclusion of a large number of individuals improved the precision of study estimates. Therefore, the results obtained here reflect the performance of ICT in the operational setting.

There were some limitations in the conduct of the study. The evaluation was performed using microscopy as a gold standard, fully aware of its limitations in detecting low parasite densities [32]. To improve accuracy of readings, two microscopists read all slides, blinded to each other's reading. A third reading was done to determine a final reading for all discordant field results. It is likely that some participants were included in the cross-sectional component of the study with circulating persistent antigenemia. This may have led to a reduced specificity because HRP2 antigens were detected for patients who may have successfully eliminated their parasites. This effect could have been overcome by using PCR to detect very low parasite densities that may have been missed by microscopy. However, this study was designed to evaluate operational accuracy of ICT where such cases are impossible to distinguish from current infections when using RDTs alone. It is assumed that there was no loss of quality in the RDTs as result of temperature fluctuations during transportation and storage. The storage temperature ranged between 22–29°C during the study period, albeit the single spike to 38°C recorded during transportation. These readings are within the safe temperatures for HRP2-based kits as reported in two previous studies [5,36].

The study team neither observed therapy nor assessed for adherence to medication for the children during the longitudinal component. Poor adherence to treatment may have led to recurrent parasitaemia as a result of treatment failure.

RDTs devices are new technologies that resemble HIV test kits formats previously used in this setting. During the follow-up, a significant number of patients that had consented to participate failed to honour all the scheduled appointments. Only three patients formally withdrew consent mentioning that their families were suspicious that these were actually HIV studies. It is likely that others may have kept away from the study with similar misinformation. All patients' data collected during the evaluation of persistent antigenicity was utilized to mitigate any effect (bias) caused by loss to follow-up.

## Conclusion

In conclusion, this study shows that ICT is an accurate test for diagnosis of malaria in an operational setting where microscopy is not available. It is a reliable test in detecting *P. falciparum *infection in a highly endemic region of Uganda. However, persistent antigenicity reduces the accuracy of this and other HRP2-based RDTs. The low specificity continues to be of concern, especially in children below five years of age, if they return with symptoms within four weeks of previous antimalarial treatment. The study highlights important practical issues that need consideration when using RDTs for diagnosis. Good clinical skills are essential to interpret test results given the persistent antigenicity and specificity variation for age in endemic areas. There is a need to explain to the end user the best way to use the tools for diagnosis.

## Competing interests

The authors declare that they have no competing interests.

## Authors' contributions

HC, JKT, JBR and DJK participated in designing the study. DJK and HC directed the field study. GWO participated in the data acquisition and the on-site supervisor. DJK and HC verified data, DJK and JKT analysed the data. DJK and HC participated in the manuscript preparation. All authors read and approved the final manuscript.
